# Big data and AI for gender equality in health: bias is a big challenge

**DOI:** 10.3389/fdata.2024.1436019

**Published:** 2024-10-16

**Authors:** Anagha Joshi

**Affiliations:** ^1^Computational Biology Unit, Department of Clinical Science, University of Bergen, Bergen, Norway; ^2^Department of Biotechnology, Bhupat and Jyoti Mehta School of Biosciences, IIT Madras, Chennai, India; ^3^Center for Integrative Biology and Systems Medicine, Wadhwani School of Data Science & Artificial Intelligence, IIT Madras, Chennai, India

**Keywords:** women's health, sex and gender, machine learning, artificial intelligence, biomarkers, bias

## Abstract

Artificial intelligence and machine learning are rapidly evolving fields that have the potential to transform women's health by improving diagnostic accuracy, personalizing treatment plans, and building predictive models of disease progression leading to preventive care. Three categories of women's health issues are discussed where machine learning can facilitate accessible, affordable, personalized, and evidence-based healthcare. In this perspective, firstly the promise of big data and machine learning applications in the context of women's health is elaborated. Despite these promises, machine learning applications are not widely adapted in clinical care due to many issues including ethical concerns, patient privacy, informed consent, algorithmic biases, data quality and availability, and education and training of health care professionals. In the medical field, discrimination against women has a long history. Machine learning implicitly carries biases in the data. Thus, despite the fact that machine learning has the potential to improve some aspects of women's health, it can also reinforce sex and gender biases. Advanced machine learning tools blindly integrated without properly understanding and correcting for socio-cultural sex and gender biased practices and policies is therefore unlikely to result in sex and gender equality in health.

## 1 Introduction

Women's health encompasses many aspects of physical, mental, and social wellbeing for women. Medicine has traditionally been and continues to be practiced using male body as a model system and assumes females differing mainly in the reproductive organs than males, with gynecological and reproductive health as the primary indicators of women's health. Sex hormones have far reaching impact on female (patho)physiology, well beyond reproductive system (Lauretta et al., 2017). Women's health issues therefore encompass diverse pathologies and vary depending on their life stage, such as adolescence, adulthood, and older age. Furthermore, women's health is influenced by biological, environmental, and social factors that may differ from those affecting men.

**Figure 1:**
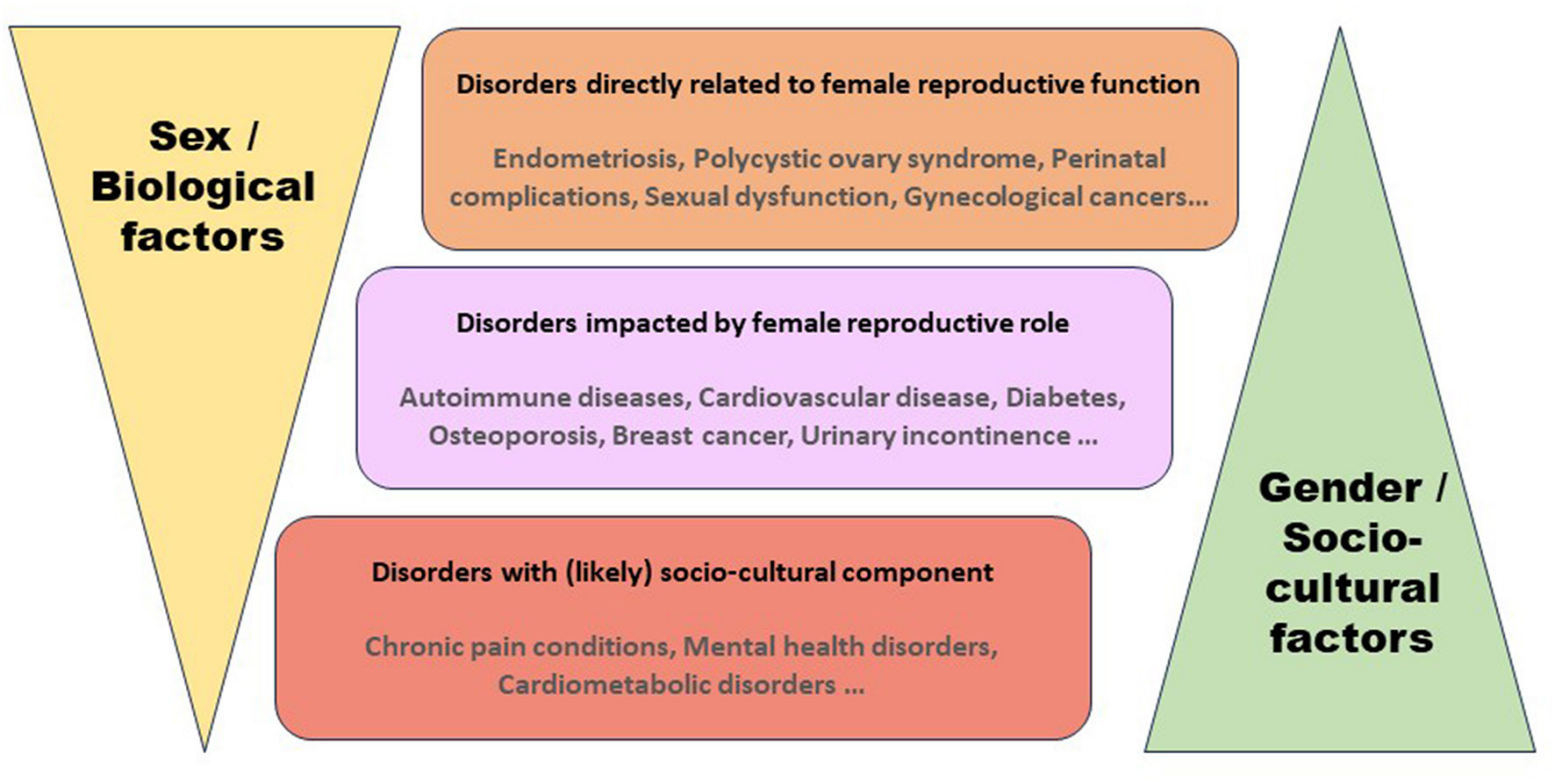
Role of biological sex and socio-cultural factors in female health.

In this perspective, pathologies are stratified in three sections (Figure 1). The first section consists pathologies that concern directly with female reproduction during female reproductive lifespan (menstruation, pregnancy, childbirth, menopause), contraception, infertility, endometriosis, polycystic ovarian syndrome, and sexual dysfunction. This category represents pathologies specific to women but have implications to progeny (both male and female). Barker (1986) performed a seminal work examining the geographical relation between ischemic heart disease mortality rates and infant mortality rates and further came up with the developmental origins of disease theory. This work was followed by many other epidemiological and genome-wide studies demonstrating unfavorable prenatal conditions can increase the risk of developing non-communicable diseases later in life, highlighting the importance of prioritizing perinatal health as a preventive strategy for lifelong health of both the mother and the progeny. The second category includes pathologies of non-reproductive organs with a sex and gender difference. Gender is defined as an individual's psychological makeup and behavior, while sex is used to refer to physical traits (Muehlenhard and Peterson, 2011). Many biological mechanisms underlie the sex and gender specific differences, such as sex hormones, cellular mosaicism, genes escaping X chromosome inactivation, and miRNAs encoded on the X chromosome (Migliore et al., 2021). Sex and gender associated diseases includes cardiovascular disease, diabetes, osteoporosis, breast cancer, depression, dementia, urinary incontinence, and autoimmune diseases. Autoimmune diseases affect females more often as female immune system generally responds more efficiently to pathogens, but also leads to over-reactive immune responses that cause more autoimmune diseases. Biomedical research has traditionally used male cell lines and subjects as females were considered more variable. The National Institutes of Health enforced in 1993 to include women in clinical research. Simply adding females in clinical studies does not reveal the role of sex and gender in physiological, behavioral, and psychological traits. Many of the current studies lack even basic sex and gender analyses such as adequate numbers of both sexes and reporting sex-disaggregated data (Kim et al., 2021). The third category represents the sex and gender differences in pathologies rooted in the socio-cultural factors. This concerns issues including gender-based violence, discrimination, poverty, education, employment, family responsibilities, and access to health care, which in turn affect nearly all pathologies. The correlation between several behavioral, psychological, and social characteristics and biological sex makes it difficult to discern the relative contributions of sex and gender to the reported sex and gender disparities in health including age of onset, prevalence, severity, symptoms, or response to medication. Despite an exponential increase recently in the number of studies characterizing sex and gender differences (over 50,00 only in last 5 years according to PUBMED search Nov. 2023), many studies lack robustness and consistency. Identification of consistent sex and gender differences across pathologies using meta-analyses (Torquati et al., 2019) is therefore of utmost importance. It is also important to publish negative results with no significant differences between men and women (Peng et al., 2022).

Artificial intelligence and machine learning is a fast-growing discipline and following successes from a variety of other disciplines, clinical researchers and practitioners are becoming increasingly interested in machine learning techniques (Sidey-Gibbons and Sidey-Gibbons, 2019). Machine learning deals with the development of algorithms to learn from data, to build predictive models resulting into efficient and accurate clinical decisions. Numerous aspects of health care, including disease management, prevention, diagnosis, and treatment, have potential for the application of machine learning. Accordingly, applications of algorithms are being developed for the healthcare industry are growing fast, ranging from automating administrative duties to analyzing medical images and genomic data to detect abnormalities, classify tumors, identify mutations, and recommend treatments to help diagnose various diseases and conditions that affect women, such as breast, cervical, ovarian cancer, endometriosis, and perinatal complications. Machine learning application can also provide personalized resources, support, and interventions to help identify and prevent the risk factors affecting women's physical and mental health. Machine learning was able to predict the preserved cognitive function of women based on various predictors such as age, education, depression, optimism, physical function, sleep disturbance, blood pressure, hemoglobin, and blood glucose (Casanova et al., 2021).

## 2 Big data and machine learning for female reproductive health

Female reproductive disorders can be broadly classified into two groups. The first being, pathologies associated with menstrual cycle. Polycystic ovarian syndrome (PCOS) is a common hormonal disorder with irregular periods, excess hair growth, acne, weight gain, infertility, and other health problems, affecting about 10% of women and has lifelong health implications. PCOS is often diagnosed by clinical symptoms, blood tests, and ultrasound scans. However, these methods can be time-consuming, expensive, and inaccurate. Thus, due to diagnostic difficulties, delayed diagnosis, and less-than-optimal treatment plans, both clinicians and patients are dissatisfied with available diagnosis and treatments options (Hoeger et al., 2021). Danaei Mehr and Polat (2022) demonstrated that ensemble machine learning algorithm can achieve a very high accuracy and sensitivity in predicting PCOS. Cessation of monthly menstruation is marked by menopause usually around the age of 50 as a result of decreased ovarian follicular activity. Over 50% of women experience hot flashes, night sweats, or both during the menopausal transition, and over 50% experience genitourinary symptoms (Crandall et al., 2023). Hot flashes can significantly lower quality of life and have been linked to Alzheimer's disease, and heart disease (Lee et al., 2022). Wearable devices can track physiological data including body temperature, sweating, activity level, and heart rate continually and recognize early warning signs of a developing hot flash. Postmenopausal women are at higher risk of osteoporosis. Indeed machine learning models with a high predictive power to help primary care physicians may be able to better identify, prevent, and treat osteoporosis early on by stratifying their patients (Shim et al., 2020).

The second group of female reproductive disorders is perinatal complications. More than half of maternal deaths globally are caused by hemorrhage, hypertensive diseases, and sepsis with direct obstetric causes accounting for about 73% of all maternal deaths (Say et al., 2014). To decrease maternal mortality by identifying high-risk pregnant women, machine learning models can identify women who are most likely to experience perinatal difficulties and recommend the best course of action by monitoring them throughout their pregnancies and utilizing information from wearable technology, social media posts, and electronic health records (Clapp et al., 2021). Machine learning approaches have proven validity to predict pregnancy outcomes such as the mode of delivery, predicting perinatal problems such preeclampsia, gestational diabetes, fetal growth restriction, and preterm birth, and other possible complications during pregnancy (Bertini et al., 2022). A systematic review of 26 studies found that machine learning algorithms can achieve high accuracy and performance in predicting pregnancy outcomes, using various features such as maternal age, weight, blood pressure, fetal heart rate, and ultrasound measurements (Islam et al., 2022). In order to give personalized and dynamic alternatives to traditional labor charts, a study that analyzed over 200,000 deliveries using artificial intelligence produced a labor risk score that predicts a composite of unfavorable birth, maternal, and neonatal outcomes as labor progresses (Shazly et al., 2022). A recent review of machine learning in perinatal care provided specific guidelines toward developing practical and helpful machine learning-based clinical decision support systems that expectant mothers and medical professionals can use, improving dataset accessibility, uncovering the unknown causes of maternal complications, and investigating the possibilities of surgical robotic tools (Islam et al., 2022). Importantly, there have been rare success stories of clinical implementation as well. A deep learning-based algorithm has been implemented by the National Health Service in Britain to assess ultrasound images obtained during a woman's 12-week scan and provide a risk score for unfavorable pregnancy outcomes, including preeclampsia, stillbirth, and fetal growth restriction. Perinatal mental health issues can also be treated or prevented with the help of new technology and devices. A virtual reality system, for instance, can assist a user overcome trauma or fear by exposing them to a simulated delivery situation (Novick et al., 2022).

## 3 Sex and gender stratified medicine and biomarker discovery

Sex stratified medicine aims to improve the safety and efficacy of treatments by accounting for differences of disease manifestation and treatment between men and women in the design and analysis of clinical trials and other studies. Biomarkers remove the subjectivity of the medical professional by offering an objective indicator for patient stratification, precision prognostics, and precision drug administration (Reel et al., 2021). Proteomic biomarkers have proven powerful to detect gestational hypertension and preeclampsia prior to clinical manifestation (Chen et al., 2011). Machine learning models using plasma proteomic data predict spontaneous preterm delivery with intact membranes more accurately and sooner in pregnancy (Tarca et al., 2021). A very high accuracy was achieved by a machine learning-based model for preeclampsia risk from multiomics datasets of cohort of pregnant women (Maric et al., 2021). Over half of obstetrical problems were correctly predicted by a recent machine learning model on electronic medical record data from 300,000 deliveries (Escobar et al., 2021). Urushibara et al. (2022) classified the endometrial tissue photos into normal, hyperplasia, and malignancy using a variety of deep learning models. Wang et al. (2022) developed a diagnostic prediction model for endometrial cancer by combining three of the best machine learning techniques with nine clinical characteristics. Mao et al. (2022) developed an automated staging model for early endometrial cancer based on deep learning and MRI data with high accuracy, lowering the rate of radiologists misdiagnosing patients. Takahashi et al. (2021) used deep learning and hysteroscopy to diagnose endometrial cancer, which outperformed human specialists in autonomously detecting and classifying the endometrial lesions.

## 4 Gender perspectives, socio-cultural factors

Sex and gender inequalities might originate from true biological differences or from social injustices as sex and gender bias does not originate from a single source. Biological health disparities can be generated, suppressed, or strengthened by behavioral, psychological, personal, cultural, and societal variable. Gender-related experiences and behaviors, such as nurturing, competitiveness, and sexual activity, affect the biological aspects of sex, such as sex hormone levels. Azizi et al. (2022) used machine learning to examine the role of sex and gender factors in COVID-19 test positivity and hospitalization and found that high-risk jobs, crowded living arrangements, and living in deprived areas were associated with increased COVID-19 infection in females, while high-risk cardiometabolic characteristics were more influential in males. Compared to men, women live longer. This is partly explained by biological difference as estrogen and other female-specific lifespan expanding genes (Vina et al., 2011), and partly gender difference as females are more likely to take preventive measures, seek medical help and adopted behavioral changes (Chen et al., 2016). Women thus live longer but in a worse physical condition i.e. frailer (Tazzeo et al., 2023). This gap might be explained by the allocation of power between men and women in social, political, and educational institutions i.e. institutionalized gender, which also creates social norms that define, uphold, and frequently justify disparities in opportunities and expectations between men and women. For example, Naganathan and Sambrook (2003) observed that bone mineral density is similar in males and females implying that the gender variations in osteoporotic fracture incidence and bone fragility must be explained by other factors. Furthermore, being regarded as a man or a woman elicit distinct responses, clinicians may therefore diagnose and recommend interventions differently based on gender. Therefore, the utilization of preventative measures and the acceptance or referral of invasive therapy strategies are strongly determined by gender. Women carry disproportionate burden of metal health related issues including depression, anxiety, post-traumatic stress disorder, and suicidal tendencies (Zelco et al., 2023), and can have negative impacts on the wellbeing of women, their children, and their families. Wearable technology, or wearable electronics such as smart watches, bracelets, and rings, can track physiological signals like skin conductance, blood pressure, and heart rate, which has shown promise to act as reliable proxies for monitoring changes in stress levels or mood (Novick et al., 2022). Another promising avenue is online consultation platform where users can benefit from self-help modules, exercises, feedback, or support offered by internet-based cognitive behavioral therapy. For instance, an internet-based cognitive behavioral therapy program can teach the user how to challenge their negative thoughts and practice positive coping skills (Redshaw and Wynter, 2022). In a number of diseases, gender-related behaviors influence risk exposure and preventative measures (Mauvais-Jarvis et al., 2020). Heart disease is more common in men than women, yet more women than men pass away within a year after suffering a heart attack. This is likely because women are disadvantaged at all stages of diagnosis and management of cardio-metabolic diseases (Kononenko, 2001). The differences between men and women in terms of the epidemiology, manifestation, pathophysiology, treatment, and disease outcomes, cardiovascular diseases, including heart failure, pressure overload, hypertension, coronary artery disease, and cardiomyopathy, cardiovascular diseases are one of the best studied systems. Numerous animal models have been used to study corresponding sex differences, and mechanistic studies have been conducted to examine the found sex differences (Regitz-Zagrosek and Kararigas, 2017). A machine learning approach created a gender index using principal component analyses and logistic regression, and to determine the association between gender, sex, and cardiovascular risk factors among patients with premature acute coronary syndrome. Half of women in the study had a masculine gender score, and 16% of men exhibited a feminine gender score. Thus, traditional sex differences in cardiovascular disease risk factors may be partly explained by patient's gender-related characteristics (Pelletier et al., 2015).

## 5 Discussion

The dominant sex and gender in society has been and still is male. Medical research has been typically done by men, on men, for men and the results are applied to both men and women. Other than the reproductive organs, many body organs function differently in men and women, and this difference is not meaningfully accounted for in medicine. Drug metabolism also varies between sexes due to differences in body composition. Eighty percent of drugs withdrawn from the market are due to their side effects in females. This is mainly because most drugs have been tested on male cells and male animal models. A recent study noted that though the percentage articles that separated sex of the cells analyzed increased, male bias and sex omission were still frequently observed. Even when both male and female cells were employed in the research, the data were rarely analyzed according to sex (Kim et al., 2021).

Machine learning can potentially offer improved detection and diagnosis by reducing the cost and time, improving the accuracy and reliability of diagnosis, providing early detection and intervention, enhancing patient care and satisfaction and facilitating personalized treatment and management. Furthermore, it has the potential to revolutionize the healthcare system and empower women to take charge of their own health. However, a lot of careful consideration is needed before automated computational methods can be widely adopted in clinical detection and diagnosis. Major considerations need to be given to technical issues such as data quality, security, interoperability, and scalability and human ethical issues such as user acceptance, engagement, satisfaction, ethics, bias, and explainability.

**Figure 2:**
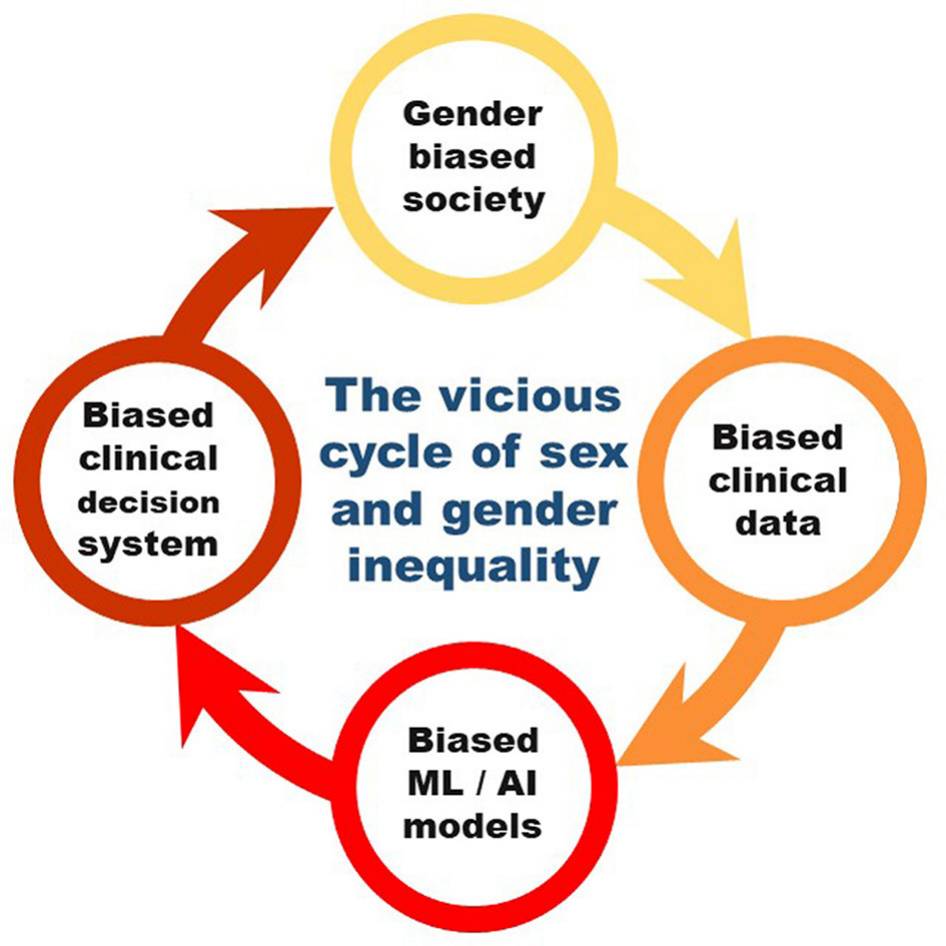
The vicious cycle of sex and gender inequality perpetuated via machine learning in clinical practice.

Figure 2 represents potential issue of implementation of AI without careful consideration in clinical practice. The algorithms carry and even exaggerate the biases in the society. Women are disadvantaged by discrimination rooted in socio-cultural factors for a variety of reasons, including unequal power relationships between men and women, social norms that limit opportunities for education and paid work, an emphasis solely on women's reproductive roles, and the possibility or experience of physical, sexual, or emotional abuse (Figure 2, yellow). Resolving centuries-old injustices resulting from a patriarchal healthcare system is the goal of women's health. For example, there is a higher likelihood of persistent pain in women. Treatment is impacted by gender preconceptions; women are given less painkillers and must wait longer for care. Men tend to be treated more seriously than women when they complain of pain, which is a reflection of ingrained social biases. Societal biases are also reflected in medical practice (Figure 2, orange). The race-correction in spirometers is one of the very well-studied examples of systematic biases in medical profession. The race and ethnicity-specific correction factors for spirometers were established on observed lung capacity differences between race and ethnicity were established in 1999. Assuming innate biological differences, racial correction was commonly incorporated into the software of spirometers (Hankinson et al., 1999). This practice of racial discrimination continued for decades before a final report in 2021 in the US, to put an end to the misapplication of race in clinical decision support systems, such as spirometry for pulmonary function assessment.

In conclusion, discrimination against women in the medicine has a long history. Biases in the data are implicitly carried by machine learning. Diagnostic algorithms and practice guidelines that modify or “correct” their outputs based on a patient's race/gender are one covert way that inequality is introduced into medicine and thereby further exaggerated in the society. The algorithms used by doctors to personalize risk assessment and direct treatment choices, are the very same algorithms that advance race and gender inequalities (Vyas et al., 2020). Many algorithms are have limited interpretability, making it challenging to identify AI bias. AI bias may originate from the algorithm's designers or from the data that was used to train it. As a result, female patients may suffer as a result of AI health applications inheriting this injustice from their data sources (Figure 2, red). Straw and Wu (2022) observed that machine learning algorithm was more likely to miss females. Thus evaluating biases in the initial stages of machine learning is critical to prevent the digitisation of inequalities into algorithmic systems (Straw and Wu, 2022). Improving women's health outcomes requires addressing gender inequities, and research should both focus on the various societal determinants that differ between men and women to explore the feasibility and effectiveness of automation in real-world settings (Redshaw and Wynter, 2022). Additionally, more collaboration is needed between researchers, clinicians, patients, regulators, and other stakeholders including women of ethnic and cultural backgrounds in the design and development of machine learning solutions for women's health to ensure the safe and responsible use of machine learning and AI. Professionals may have personal unconscious prejudices that influence the programs they create. More diversity in AI could aid in lessening this issue, however women are currently the minority in machine learning and AI. Full understanding of biases in the data and further correcting for them is nearly missing. Thus, it is unlikely that the integration of cutting-edge machine learning techniques with existing ideas, practices, and regulations will result in sex and gender health equality.

## Data Availability

The original contributions presented in the study are included in the article/supplementary material, further inquiries can be directed to the corresponding author/s.
